# Does patenting always help new firm survival? Understanding heterogeneity among exit routes

**DOI:** 10.1007/s11187-021-00481-w

**Published:** 2021-05-25

**Authors:** Masatoshi Kato, Koichiro Onishi, Yuji Honjo

**Affiliations:** 1 School of Economics & Research Center for Entrepreneurship, Kwansei Gakuin University, Hyogo, Japan; 2 School of Education, Waseda University, Tokyo, Japan; 3 Faculty of Commerce, Chuo University, Tokyo, Japan

**Keywords:** Patent, New firm survival, Exit route, Bankruptcy, Merger, Voluntary liquidation, L26, M13, O30

## Abstract

**Abstract:**

While patents are a valuable resource ensuring the competitive advantage of firms, there is limited evidence on the role of patents in the survival and exit strategies of new firms. To fill the gap in the literature, we examine whether the effects of patenting on new firm survival vary according to exit routes (bankruptcy, merger, and voluntary liquidation), while considering the endogeneity of patenting. We use a large-scale sample of new firms in the Japanese manufacturing and information services sectors for the period 2003–2013. The findings indicate that new firms with a higher stock of patents are less likely to go bankrupt. Conversely, new firms with a higher stock of patents are more likely to exit via merger. These findings are consistent, regardless of whether patent stock is measured based on the patent applications or granted patents. Furthermore, we provide evidence that new firms with a higher stock of granted patents are more likely to voluntarily liquidate their businesses.

**Plain English Summary:**

Can new firms enjoy a “patent premium” in terms of survival and exit outcomes? The findings of this study indicate that (1) patenting reduces the risk of bankruptcy, and (2) it increases the odds of exit via merger and voluntary liquidation. On the one hand, patenting ensures that new firms obtain competitive advantages, and thus, survive in the product market. On the other hand, it enables new firms to pursue successful exit strategies in the markets for ideas. This study concludes that new firms can enjoy a patent premium in terms of survival and exit outcomes. In promoting sustainable economic growth via entrepreneurship, policymakers need to shift their focus from creating more firms to creating innovative firms.

## Introduction

New innovative firms play a vital role in driving innovation and economic growth (Wennekers & Thurik, 1999; Aghion et al., 2009; Block et al., 2013; Bos & Stam, 2014), especially in modern entrepreneurial economies (Thurik et al., 2013). Meanwhile, it is well recognized that many firms exit some years after their foundation (Bartelsman et al., 2005) in different ways (Headd, 2003; Wennberg et al., 2010; Cefis & Marsili, 2011, 2012; Coad, 2014). While some firms are forced to exit because of business failure, others close voluntarily for various reasons (Harada, 2007). Moreover, some firms exit via merger and acquisition (M&A) as a desirable option (DeTienne & Cardon, 2012; Cotei & Farhat, 2018). While some scholars have addressed the exit route of new firms (e.g., Grilli et al., 2010; Cefis & Marsili, 2011, 2012; Kato & Honjo, 2015; Ponikvar et al., 2018), a research gap still exists in understanding the antecedents of new firm survival according to exit routes, such as bankruptcy, M&A, and voluntary liquidation.

To date, numerous studies have highlighted the role of firms’ innovative capabilities, a major source of competitive advantage in determining firm survival (Cefis & Marsili, 2005, 2006; Colombelli et al., 2016).^1^ In practice, successful innovation enables firms to increase their probability of survival and has a stronger impact on new firms than on large, established ones (Rosenbusch et al., 2011). Using patents is a well-known, significant innovation strategy to protect inventions and attract customers and external providers of capital (Audretsch et al., 2012; Holgersson, 2013; Zhou et al., 2016). The importance of the strategic use of intellectual property (IP) is increasing for technology-based firms to gain competitive advantage (Cho et al., 2018). However, acquiring and enforcing IP rights is costly, especially for small firms (Lanjouw & Schankerman, 2004; Jensen & Webster, 2006). Hence, it is still unknown whether patenting helps new firms survive or exit successfully.

To fill the gap, this study examines the role of patenting in determining new firm survival according to exit routes. We construct a large-scale sample of new firms in the Japanese manufacturing and information services sectors by matching a credit-reporting database with a Japanese patent database. In particular, we examine how the effects of patenting on new firm survival vary across exit routes (bankruptcy, merger, and voluntary liquidation), while taking into account the endogeneity of patenting. The major findings of this study are summarized as follows. First, new firms with a higher stock of patents are less likely to go bankrupt, regardless of whether patent stock is measured based on applied or granted patents. Second, new firms with a higher stock of patents are more likely to exit via merger, regardless of which patent measures are used. Third, new firms with a higher stock of granted patents are more likely to voluntarily close their businesses, while a stock of patent applications is not significantly related to voluntary liquidation. This study contributes to a better understanding of the role of patenting in new firm survival by identifying heterogeneity in the effects of patenting among exit routes.

The remainder of this paper is organized as follows. Section 2 discusses the theoretical background of this study and develops hypotheses. Section 3 explains the empirical model used, including the estimation methods and variables. Section 4 describes the data used in the analysis and shows some descriptive statistics, while Section 5 presents the results and conducts robustness checks. Section 6 summarizes the findings and discusses the implications and limitations.

## Background and hypothesis development

### Divergent exit routes

To date, numerous studies have examined firm survival as post-entry performance (e.g., Audretsch, 1991; Mata & Portugal, 1994; Audretsch & Mahmood, 1995; Honjo, 2000). While many of these studies treated exits as homogenous events, recent research emphasizes divergent exit routes (e.g., Wennberg et al., 2010; Coad, 2014; Wennberg & DeTienne, 2014). While some firms are forced to exit because of business failure, others plan their exits when their businesses are performing well. In practice, there are many exit options available to new firms.

Some studies distinguish between firm liquidation and sale as an exit route (e.g., Coad, 2014; Kato & Honjo, 2015). Wennberg et al. (2010) indicated four types of exit: harvest liquidation, distress liquidation, harvest sale, and distress sale. Wennberg et al. (2010, p. 364) define harvest sale as the sale of a high-performing firm that continues operations after the entrepreneur exits as a majority owner, while a distress sale is the sale of a firm in financial straits. As M&A exits, regarded as a typical form of firm sale, are desirable outcomes for some business owners, it is worth identifying which factors explain the M&A exit outcomes (Cotei & Farhat, 2018). Though distressed firms either decide to exit voluntarily or are forced into bankruptcy, voluntary liquidation fundamentally differs from bankruptcy (Balcaen et al., 2012). Coad (2014, p. 723) indicated that voluntary liquidation occurs when businesses fail to be viable economic entities or when the entrepreneurs consider other outside options. Voluntary liquidation can be characterized as “relatively unviable,” usually occurring when a business is failing or otherwise seen as unviable. Meanwhile, Harada (2007) emphasized differences between economic-forced and non-economic-forced exits (e.g., aging of a manager and despairing perception of further business) for small firms. Voluntary liquidation is an option irrespective of business performance (Wennberg et al., 2010; Coad, 2014). In these respects, there should be sharp differences between exit routes; however, without distinguishing between them, we may misinterpret the nature, determinants, and consequences of firm exit, including failure and non-failure outcomes.

Table 1 reviews empirical studies on exit routes. Schary’s (1991) early work on exit routes, which examined different exit routes in the cotton textile industry, assumed that they should be inherently ordered as survival, merger, non-failure, and failure. Other studies examining firm exit have differentiated closure from M&As (Fontana & Nesta, 2009; Grilli et al., 2010; Cefis & Marsili, 2011, 2012; Esteve-Pérez et al., 2010). Still others have grouped closures into bankruptcy and voluntary liquidation (Harhoff et al., 1998; Mata et al., 2007; Balcaen et al., 2012; Kato & Honjo, 2015; Ponikvar et al., 2018). Among these exit studies, Fontana and Nesta (2009), Grilli et al. (2010), Wennberg et al. (2010), Balcaen et al. (2012), Cefis and Marsili (2012), Kato and Honjo (2015), and Honjo and Kato (2019) focused on new firms, showing sharp differences in the determinants of firm exit between the routes.

**Table 1:** Review of empirical studies on distinct exit routes

Author	Exit route	Major determinants	Sample	Method
Balcaen et al. (2012)	B, M, V	Firm (cash flow, leverage, debt, age), Industry (dummies)	6118 firms (firms less than 5 years old excluded), 1998–2000, Belgium.	Binomial nested logit
Buehler et al. (2006)	B, M (S)	Firm (size, age), Region (dummies), Industry (dummies)	54,750 firms (13.6 employees), 1995–2000, Switzerland.	Continuous-time duration
Cefis and Marsili (2011)	C, M (S)	Firm (product and process innovation, size, group affiliation, patent application, entrepreneurial-firm dummy), Industry (low or high-tech dummy)	3203 firms (young ones within 5 years: 8%), 1996–2003, the Netherlands.	Multinomial logit
Cefis and Marsili (2012)	C, M, R (S)	Firm (product and process innovation, age, size), Industry (Pavitt’s categories)	3275 firms (118.2 employees, 28.7 years), 1996–2003, the Netherlands.	Multinomial logit and cloglog.
Cotei and Farhat (2018)	M (S)	Founder (education, work experience, gender), Firm (positive employment growth, serial entrepreneur, R&D, dummy for patent, trademark or copyright), Industry (dummies)	3140 firms (created in 2004), 2005–2011, the USA.	Multinomial logit
Esteve-Pérez et al. (2010)	C, M (S)	Firm (size, age, labor productivity, price-cost margins, R&D, advertising), Industry (low, medium or high-tech dummy)	2998 firms (257.5 employees, 23.9 years), 1990–2000, Spain.	Continuous-time duration
Fontana and Nesta (2009)	C, M (S)	Firm (technology frontier, R&D intensity, size, age)	121 firms (470–480 employees), 1990–2005, worldwide LAN switching industry.	Multinomial logit and cloglog
Grilli et al. (2010)	C, M (S)	Firm (size, age), Industry (dummies)	13,574 firms (1 or 2 employees, 0 to 13 years), 1983–2006, Italy.	Continuous and discrete-time duration (cloglog)
Harhoff et al. (1998)	B, V (S)	Firm (size, ownership, diversification, legal status), Industry (dummies)	10,902 firms (276 employees, 19 years), 1989–1994, West Germany.	Continuous-time duration
Honjo and Kato (2019)	B, M (S)	Firm (initial debt finance (size/ratio), initial equity finance (size/ratio), dummy for minimum capital requirement regulation), Industry (dummies)	16,185 firms (joint-stock companies, less than 100 employees), 1995–2011, Japan	Continuous-time duration
Kato and Honjo (2015)	B, M, V (S)	Founder (educational level/field, age, gender), firm (paid-in capital, age, legal status), Region (unemployment rate), Industry (HHI, growth, capital intensity, low- vs. high-tech sector)	7868 firms (less than 100 employees, 0 to 12 years), 1997–2004, Japan.	cloglog and random-effects cloglog
Mata et al. (2007)	B, V (S)	Firm (size, age, debt, bank relationship, foreign ownership, worker’s wages, worker’s schooling), Industry (dummies)	413,586 observations (14.8 years), 1995–2000, Portugal.	Multinomial logit
Ponikvar et al. (2018)	B, M, V (S)	Firm (financial conditions, size, age, labor productivity, fixed asset ratio), region (dummies), Industry (dummies)	55,810 firms (7.8 employees, 10.2 years), 2006–2012, Slovenia.	Multinomial probit
Schary (1991)	B, M, V (S)	Firm (debt, cash flow, other financial characteristics)	61 firms, 1924-1940, New England textile industry.	Ordered logit and multinomial logit
Wagner and Cockburn (2010)	D, M (S)	Firm (age at IPO, size, total assets, patent application, patent citation), Industry (dummies)	356 firms, (5.9 years), 1998–2005, US Internet-related industries.	Continuous-time duration
Wennberg et al. (2010)	Harvest/distress liquidation and sale, (S)	Founder (Entrepreneurial/industry experience, education, gender, outside job, age), Firm (size, ownership by the parent firm, reinvestment), Industry (dummies)	1735 firms (3 employees, 4.5 years), 1995–2002, Sweden.	Multinomial logit

Note:(1) *B* bankruptcy, *M* mergers and/or acquisitions, *V* voluntary liquidation, *C* closure, *R* restructuring, *D* divestiture, *S* survival. (2) S in parentheses—(S)—means that the base outcome of the estimated model is “survival.” (3) Firm, Industry, and Region indicate firm-, industry-, and region-specific variables, respectively. (4) In the fourth column (“Sample”), the sample average in firm size (number of employees) and/or firm age (years after start-up/foundation) are presented in parentheses

### Patenting and exit routes

Based on the resource-based view of the firm (RBV), it is well recognized that patents are important resources that create a unique competitive advantage for firms (e.g., Hsu & Ziedonis, 2013). Patents prevent competitors from utilizing the protected inventions for a certain period, so that firms can appropriate the returns from their investment in R&D (e.g., Levin et al., 1987). Patenting can improve firms’ competitive position, which results in a higher probability of survival (e.g., Cefis & Marsili, 2005, 2006).

Not surprisingly, new firms that lack complementary assets, such as marketing channels and production facilities, often face difficulties in appropriating returns from their innovations (e.g., Teece, 1986; Colombo et al., 2006). For small, start-up ventures, patents may be a relatively effective means of appropriating R&D returns, in part because other means such as investment in complementary sales and service efforts may not be feasible (Levin et al., 1987, p. 797). Meanwhile, new firms may have various motives for patenting. Some firms use patents as “bargaining chips,” which can improve their own position in negotiations with partners for technology access (e.g., Hall & Ziedonis, 2001; Blind et al., 2009). New firms can earn greater profits through cooperation rather than competition with existing firms through licensing, joint venture, or acquisition (Veugelers & Schneider, 2018). Negotiations about mergers, license contracts, or research co-operations depend mainly on how the partners evaluate the research efforts and results of their counterparts, which is mainly measured in the number of patents in the companies’ portfolio (Blind et al., 2009, p.429). As the extreme case, the markets for “ideas” may operate through acquisitions of new innovative firms by established firms (Blonigen & Taylor, 2000; Gans & Stern, 2003).^2^ Such “division of innovative labor” is closely related to growing technology markets in high-tech industries (Arora et al., 2001; Veugelers & Schneider, 2018).

In reality, new innovative firms are often targeted by large established firms to acquire patents as a valuable resource. For example, Apple Inc. acquired NextVR Inc., a new three-dimensional virtual reality content transmission programming service provider, with a number of patent rights in this field, in a deal valued at approximately 100 million US dollars in May 2020. It is often argued that M&A markets in Japan are not well developed (Honjo & Nagaoka, 2018; Honjo, 2020). According to a report by Recof Data Corporation, a company collecting M&A information in Japan, the number of M&As involving Japanese firms increased twofold in two decades. Nowadays, new innovative firms are often merged or acquired by large established firms in Japan. For example, Soracom Inc., an IoT-optimized cellular network operator founded in 2014, was acquired by KDDI Corporation (a huge cellular network operator) in 2017. According to databases compiled by Bureau van Dijk (*Zephyr* and *Orbis Intellectual Property*), this transaction includes the sale of patents held by Soracom with 18 patent families at the time of the acquisition’s announcement. Because of increasing attention to acquiring patents, it would be worthwhile investigating whether patents trigger M&As in the markets for ideas.

Table 2 shows a review of previous studies on the effect of patenting on firms’ exit routes. Among these studies, Wagner and Cockburn (2010) showed that patent applications generally reduce the probability of exit for Internet-related IPO firms listed in the NASDAQ market. They distinguished between the exit routes of merger and delisting, and found that patent applications have a significantly negative effect on exit via merger but no significant effect on delisting. In contrast, Wagner and Cockburn (2010) showed that patents with higher forward citations increased the probability of exit via merger. Cefis and Marsili (2011) examined whether patenting affects exit via closure and M&A for young firms in the Netherlands; they showed that patent applications (measured as a dummy) decrease the probability of exit via M&A but did not significantly affect the probability of closure. Using data from the Kauffman Firm Survey conducted in the USA, Cotei and Farhat (2018) found that young firms with IP rights, including patents and trademarks, are more likely to become targets for M&A.

**Table 2:** Review of empirical studies on the relationship between patenting and exit

Author	Patent measure	Major finding	Sample
Buddelmeyer et al. (2010)	(1) Patent application (2-year lagged), (2) patent stock (aggregates of number of years in-force)	(1) Positive on exit, (2) negative on exit	299,038 firms (all companies registered), 1997–2003, Australia
Cefis and Marsili (2011)	Patent application (dummy)	Negative on exit via M&A, insignificant for closure	3203 firms (young ones within 5 years: 8%), 1996–2003, the Netherlands
Colombelli et al. (2013)	Patent application stock	Positive on survival	74,862 manufacturing firms (firms created by 2001), 2001–2011, France
Cotei and Farhat (2018)	IP rights (dummy for patent, trademark, or copyright)	Positive on exit via M&A	3140 firms (created in 2004), 2005–2011, the US
Helmers and Rogers (2010)	Patent application (dummy)	Negative on exit	131,325 firms, (limited companies incorporated in 2001), 2001–2005, UK
Levitas et al. (2006)	(1) Average citation ratio (# citations adjusted by the application year and patent class), (2) patent activity (dummy)	(1) Negative on exit, (2) insignificant	295 firms (the number of employees is approximately 500), integrated circuit industry
Wagner and Cockburn (2010)	(1) Patent application (dummy), (2) # of US patent application, (3) # patents w/more than 6 cites	(1) Negative on pooled exit, merger, and delisted, (2) negative on pooled exit and merger, insignificant on delisted, (3) positive on exit via merger	356 firms (5.9 years), 1998–2005, US Internet-related industries

Until now, however, there is limited evidence on the effects of patenting on exit routes. To the best of our knowledge, there are no studies on the effects of patenting while considering its endogeneity. In addition, previous studies do not distinguish between bankruptcy (failure) and other types of closure (e.g., voluntary liquidation). In this study, we examine the effects of patenting on firms’ exit by distinguishing between bankruptcy, merger, and voluntary liquidation, while considering the endogeneity of patenting.

### Hypothesis development

Based on the above arguments, we develop our hypotheses on the effects of patenting on exit routes (bankruptcy, merger, and voluntary liquidation).^3^ Drawing on the RBV, we consider alternative commercialization strategies using patents: competitive strategy in product markets and cooperative strategy with partners in markets for “ideas.”

Firms need to secure valuable resources, such as technological knowledge, when competing in product markets, thereby gaining competitive advantage (Wernerfelt, 1984; Barney, 1991). Especially for new firms, patents are an essential resource to protect inventions and attract customers and external providers of capital (Wagner & Cockburn, 2010; Cefis & Marsili, 2011; Audretsch et al., 2012; Holgersson, 2013; Zhou et al., 2016). In addition, patenting can be an indication of a firm’s growth potential to external stakeholders (Holgersson, 2013; Cotei & Farhat, 2018). Patenting is widely regarded as entrepreneurs’ commitment to developing their innovative ideas (Cefis & Marsili, 2011). Entrepreneurs without growth ambitions are unwilling to patent inventions despite having developed ideas, since it takes time and fees to apply and request substantial examination from patent offices. Under information asymmetry, patenting apprises potential lenders and investors, including venture capitalists, of the firm’s technological capabilities (Hsu & Ziedonis, 2008; Audretsch et al., 2012; Conti et al., 2013; Hoenig & Henkel, 2015; Hottenrott et al., 2016; Veugelers & Schneider, 2018).^4^ Therefore, new firms with patents are valorized positively by external providers of capital (Zhou et al., 2016). Not only current investment in intangible assets but also previous intangible stock may affect new firm survival, since firms with a higher stock of patents have greater revenue potential (Buddelmeyer et al., 2010). As a result, firms can make commercial transactions feasible through more patents in product markets, which may ensure their competitive advantage, thereby reducing their probability of bankruptcy. For these reasons, we postulate the following hypothesis.

- **Hypothesis 1**: New firms with a higher stock of patents are less likely to go bankrupt.

While some new firms may choose to compete with existing rivals in product markets, others may intend to use their patents as a tool to negotiate with partners in markets for “ideas” (Gans & Stern, 2003). The patents held by small technology–oriented firms are their most marketable assets, providing something tangible to offer when they sell out later (Levin et al., 1987, p. 797).^5^ Generally, firms with patents, especially high-quality ones, become more attractive targets for M&A because of the market value of their ideas (Wagner & Cockburn, 2010). Scholars have argued that some patentees file for patents simply to improve their chances of being acquired (Graham & Sichelman, 2008). Established firms typically pay high premiums to acquire high-potential start-ups that could result in substantial rewards for founders (Cotei & Farhat, 2018). Meanwhile, the patent portfolio is an important aspect in the due diligence for M&A (Breitzman & Thomas, 2002). Firms’ larger portfolio in terms of the number of patents puts the firms in a better bargaining position in negotiations (Blind et al., 2009; Noel & Schankerman, 2013). Therefore, we postulate the following hypothesis.

- **Hypothesis 2**: New firms with a higher stock of patents are more likely to exit via merger.

In the markets for ideas, new patenting firms can also sell their patent rights to other organizations and thus cash out their inventions, even if they cannot find appropriate partners for merger. Voluntary liquidation can generate higher sales proceeds than a merger, if multiple acquirers can redeploy the assets into higher-valued uses than what a single acquirer can possibly achieve (Kim & Schatzberg, 1987).^6^ The probability of voluntary liquidation is higher when the expected liquidation value is higher, as well as when the expected M&A value is lower than the expected liquidation value (Balcaen et al., 2012). New firms with more patents may have an option to close their businesses with solvency by holding patents as intangible assets or selling them out, and they can expect higher liquidation value. Based on these arguments, we postulate the following hypothesis.

- **Hypothesis 3**: New firms with a higher stock of patents are more likely to exit voluntarily.

To test the above three hypotheses, in the following sections, we present the model and data used in the empirical analyses.

## Method

### Empirical model

We estimate the effect of patenting on the survival of new firms according to exit route. As shown in Table 1, while some empirical studies have used the continuous-time model to examine the duration of firm survival according to exit route (e.g., Buehler et al., 2006; Esteve-Pérez et al., 2010; Honjo & Kato, 2019), others have used the discrete-time duration model (e.g., Fontana & Nesta, 2009; Cefis & Marsili, 2011, 2012; Kato & Honjo, 2015). As the timing of survival and exit is observable only at the year level, we use the discrete-time duration model following the previous studies.

We classify exits into three routes: bankruptcy, merger, and voluntary liquidation. Let *T*_*ij*_ denote a discrete-time random variable, which represents the period when firm *i* exits via route *j* (= 1, …, *m*). To model the transition from survival to exit, we define a hazard function *h*_*ij*_(*t*), which represents the conditional probability of a transition to route *j* between periods *t* and *t*+1 for surviving firm *i*. Using a probit model, the hazard function can be expressed as follows:

1 $$ {h}_{ij}(t)=\Pr \left({T}_{ij}=t+1|{T}_{ij}>t\right)=\Phi \left({x}_{it},{\beta}_j\right), $$

where Φ(∙) is the standard normal cumulative density function, *x*_*it*_ is a vector of the covariates (some are time varying) that affect the survival and exit route of firm *i*, and *β*_*j*_ denotes the parameters to be estimated.

However, the propensity to patent is likely to be affected by various factors. For example, some studies provide evidence that industry-specific characteristics, such as appropriability conditions and technological opportunity, affect the propensity to patent (e.g., Brouwer & Kleinknecht, 1999; Ceccagnoli, 2009; Dindaroğlu, 2018).^7^ In addition, the propensity to patent may depend on a firm’s life cycle, since knowledge is cumulative in nature. Some studies showed evidence that the propensity to patent changes according to firm age (e.g., Balconi & Fontana, 2011). Therefore, patenting is considered as endogenous in the model. Estimating without taking into account endogeneity will lead to biased results. To deal with potential endogeneity, an instrumental variable (IV) probit model is the most desirable as an estimation model (Wooldridge, 2010). Therefore, in this study, we use an IV probit model to examine the effects of patenting on the survival of new firms according to exit route.

### Probability of exit routes

Bankruptcy as involuntary liquidation is the situation in which firms cannot repay their debts and thus cease operations. Bankruptcy includes firms applying for court protection under Bankruptcy Law and those under the Corporate Rehabilitation Law or the Civil Rehabilitation Law. Additionally, despite the absence of a court judgment, firms are considered to be bankrupt when banks stop providing credit to service bills payable. Thus, bankruptcy includes not only legally bankrupt firms but also economically inactive ones. Undoubtedly, the occurrence of bankruptcy depends on the bankruptcy laws in the country. As pointed out by Peng et al. (2010), in Japan, even when financially insolvent firms decide to file for bankruptcy, courts will scrutinize the case and decide whether to allow certain firms to declare themselves bankrupt which can be a lengthy process (Lee et al., 2007; Peng et al., 2010). Meanwhile, it is often argued that Japanese firms with strong bank ties are more likely to avoid bankruptcy than firms without close bank ties, since the concentration of debt and equity enables the bank to restructure the firm’s liabilities without relying on the coordinating role of bankruptcy courts (Suzuki & Wright, 1985; Hoshi et al., 1991). In addition, because the exit mechanism of insolvency is generally not profitable for firms below a certain size and an insolvency procedure involves high transaction costs, debtors and creditors may prefer less formal agreements, such as voluntary liquidation (Harhoff et al., 1998). Bankruptcy is not a reasonable option for small firms in Japan, who usually opt instead for voluntary liquidation (Harada, 2007). The variable for bankruptcy (*Bankruptcy*) is defined as a dummy variable indicating 1 if the firm goes bankrupt between periods *t* and *t*+1, 0 if the firm survives.

Merger describes the situation in which firms disappear by being combined with other firms. The situation of M&A markets differs between countries. Exit via M&A is generally regarded as successful. However, in Japan, successful exit strategies via M&A are much rarer than in the USA (Honjo & Nagaoka, 2018). In practice, M&As in Japan tend to be counter-cyclical, while US M&As are procyclical (Mehrotra et al., 2011). This may be partly because M&As are often employed to rescue financially distressed firms rather than to expand businesses during economic booms. Such “rescue mergers” occur when high-performing firms acquire important suppliers or subcontractors that are in financial distress (e.g., Kang et al., 2000; Kubo & Saito, 2012; Honjo & Nagaoka, 2018).^8^ The variable for exit via merger (*Merger*) is defined as a dummy variable indicating 1 if the firm exits via merger between periods *t* and *t*+1, 0 if the firm survives.

Voluntary liquidation describes the situation in which firms voluntarily dissolve their businesses without insolvency. Several reasons seem to exist for voluntary liquidation. While some entrepreneurs dissolve their businesses without insolvency due to poor performance, others voluntarily dissolve their businesses due to employment opportunities with high wages. Some entrepreneurs may choose to close their firms because they are approaching retirement age and have no successors. Japan, currently experiencing an aging population and declining birthrate, represents a unique context for voluntary liquidation. According to the Small and Medium Enterprise Agency (2014), the number of voluntary liquidations (e.g., company dissolution and business closure) in Japan doubled from 2003 to 2013; in the same period, bankruptcies declined by approximately 30%. The most common reasons (cited by approximately half the respondents) for voluntary liquidation were managers’ aging and health problems. Using data on small firms in Japan, Harada (2007) found that only 40% of firm exits were economically driven. The variable for voluntary liquidation (*Voluntary*) is defined as a dummy variable indicating 1 if the firm liquidates voluntarily the business between periods *t* and *t*+1, 0 if the firm survives.

We identify the exit year of new firms based on information on the accounting year of their final financial statements.

### Patent stock

In this study, patenting is the main independent variable determining the survival of new firms. It is well known that the distribution of patent values is highly skewed toward the low end (Trajtenberg, 1990). Patent citations correspond to the number of times a patent has been cited in more recent patent applications, and thus are a proxy for the importance of a patent (Markman et al., 2004). A firm with a large number of cited patents is likely to possess technology that is central to developments in its industry, and such a firm exhibits the ability to produce innovative technologies that have had a strong influence on later developments in its industry (Breitzman & Thomas, 2002). In practice, Narin et al. (1987) provide evidence on a positive relationship between highly cited patents and increased sales and profits in the pharmaceutical industry. Therefore, patent citations may represent firms’ capabilities associated with survival and exit. In addition, highly cited patents may be highly evaluated in the markets for ideas. In practice, the value of patents is investigated in the due diligence of M&A (Breitzman & Thomas, 2002). Firms with highly cited patents are a more attractive target for merger. Even if firms with highly cited patents do not find a suitable partner for merger, they can sell their patents at a high price in the markets for ideas. These firms may receive a large amount of cash in the markets for ideas, which may promote voluntary liquidation (Balcaen et al., 2012).

Following Hall et al. (2001), we capture the value of patents by considering the number of forward citations. We measure an index for citation-weighted patent application counts (*Patcite*_*it*_) for firm *i* in period *t* as follows:

2 $$ {Patcite}_{it}=\sum \limits_k\left(1+\frac{Cite_{kt}}{A\_{cite}_{lt}}\right), $$

where *k* represents firm *i*’s patent applications, and *Cite*_*kt*_ is the number of citations that patent application *k* received divided by the average citation count for a group of patents to which the patent application of interest *k* belongs (*A*_*cite*_*lt*_), that is, technological area *l* in application year *t*.^9^ The index for a citation-weighted patent count is measured by adding 1 to the number of citations divided by the average citation, then the citation-weighted patent application count is aggregated at the firm level for each application year.

As already discussed, not only current investment in intangible assets but also previous intangible stock may affect new firm survival, since firms with a higher stock of patents have greater revenue potential (Buddelmeyer et al., 2010). In addition, firms’ larger portfolio in terms of the number of patents puts the firms in a better bargaining position in negotiations (Blind et al., 2009; Noel & Schankerman, 2013), and they can sell more patent rights and thus get a larger amount of cash. For these reasons, patenting should be measured as a stock rather than as a flow. Following previous studies (e.g., Griliches & Mairesse, 1984; Hall, 1993; Hottenrott et al., 2016), we compute firm *i*’s stock of citation-weighted patent application counts (*Patst*_*it*_) in period *t* using a constant depreciation rate (*δ*) of 15% per year as:

3 $$ {Patst}_{it}=\left(1-\delta \right){Patst}_{it-1}+{Patcite}_{it}. $$

The *ex post* value of patents can be captured with the citation-weighted patent counts (Hall et al., 2005). We first measure the stock of citation-weighted patent application counts (*Patst*_*it*_) including both granted and non-granted patents. However, patentability requires novelty (and inventive steps) and utility (Nagaoka et al., 2010). In practice, about 50% of patent applications in Japan were not granted during the observation period (2016 JPO Annual Statistics Report).^10^ While patent applications have been widely used as a measure of patenting activity (Hsu & Ziedonis, 2013; Hoenen et al., 2014; Hall, 2019), the value of granted patents seems to differ from that of non-granted patents (Kline et al., 2019). In addition, firms can trade patent rights in the markets for ideas only when patents are granted. It is worthwhile identifying the value-added effects of granted patents as an intangible asset. To take into account the *ex ante* value of patents in terms of novelty, usefulness, and non-obviousness, we measure the stock of citation-weighted patents granted by the patent authority (*Patst_gr*_*it*_) measured at the time of patent application.

### Instrumental variables

In this study, patenting is considered as endogenous in the IV probit model, and some variables are used as instrumental variables. Specifically, appropriability and technological opportunity at the industry level are used as instrumental variables in this study. It has been recognized that firms have incentives to invest in R&D when they can appropriate returns from innovating (Arrow, 1962; Levin et al., 1987). In practice, the rate of patenting tends to increase under strong appropriability conditions (Dindaroğlu, 2018). While industries differ widely in the extent to which patents are effective (Cohen, 2010), interindustry differences in innovation activities tend to be persistent (Cefis & Orsenigo, 2001). The appropriability measure has been used as a variable instrumented for patent propensity in previous studies (e.g., Ceccagnoli, 2009). In addition, firms are more likely to have incentives to innovate when technological opportunity is high (e.g., Nelson & Wolff, 1997). In practice, Brouwer and Kleinknecht (1999) showed evidence that sectors with high technological opportunity tend to have a higher propensity to patent than sectors with low technological opportunity. Meanwhile, there should be heterogeneity in firm performance within industries. While firm performance (e.g., survival and exit) is contingent on the firm’s strategy (e.g., patenting), the strategy depends on industry conditions, including appropriability and technological opportunity. For these reasons, industry appropriability and technological opportunity are likely correlated with the decision of patenting, while it can be assumed that they are uncorrelated with the error term of the equation for exit routes.

Following Levin et al. (1984, 1987), appropriability (*Appro*) is defined as the extent to which the innovative outcomes can be appropriated by the innovators themselves, while technological opportunity denotes the availability of useful information for innovation. The variable for appropriability is calculated based on the survey’s scores regarding the effectiveness of nine methods of appropriation (e.g., patents to prevent duplication or to secure royalties, secrecy, lead time). The variable for technological opportunity (*Tech*) is calculated based on the survey’s scores regarding the importance of external sources of knowledge for an industry’s technological advance (e.g., suppliers, customers, competitors, government agencies and research labs). We constructed these variables in the same way as previous studies based on Japanese data (e.g., Goto & Nagata, 1997; Okamuro et al., 2011).

### Control variables

Several control variables are included in the model. First, a dummy variable for the founders’ patent applications before founding (*Pre_pat*), which represents technological experience, is included as a control variable in the model since firms managed by founders with higher levels of human capital are more likely to exhibit superior performance (Kato et al., 2015). Additionally, founders with more technological experience may have more information on potential market demand and opportunities than those with less (Gruber et al., 2013). Furthermore, we controlled for the effect of firm size (*Size*), defined as the number of employees in the first year of observation, on new firm survival because the probability of survival is found to increase with firm size (Audretsch, 1991; Audretsch & Mahmood, 1991, 1995; Geroski, 1995; Cefis & Marsili, 2011, 2012). Additionally, we include a variable for firm age (*Age_firm*), defined as the number of years from a firm’s foundation. As indicated by previous studies, survival and exit depend heavily on firm age (Evans, 1987; Geroski, 1995; Fontana & Nesta, 2009; Cefis & Marsili, 2011, 2012); specifically, firms with a longer history are more likely to survive than newer firms because of learning effects and cumulative knowledge.

Regarding founder-specific characteristics, the founder’s age (*Age_founder*) is included to control for differences in opportunity costs and risk propensity among founders (Levesque & Minniti, 2006; De Jong & Marsili, 2015). Following previous studies, founders’ educational backgrounds are considered as a determinant of firm survival (Bates, 1990; Cooper et al., 1994; Kato & Honjo, 2015); thus, dummies for founders’ educational backgrounds (*Edu* and *Edu_X*) are included in the model. Moreover, cohort dummies (eight cohorts for the different years of entry) are included. Table 3 presents the definitions of the variables.

**Table 3:** Definitions and summary statistics of variables

Variable	Definition
(Dependent variable)	
*Bankruptcy*	Dummy variable: 1 if the firm goes bankrupt between periods *t* and *t*+1, 0 if the firm survives.
*Merger*	Dummy variable: 1 if the firm exits via merger between periods *t* and *t*+1, 0 if the firm survives.
*Voluntary*	Dummy variable: 1 if the firm voluntarily liquidates the business between periods *t* and *t*+1, 0 if the firm survives.
(Endogenous variable)	
*Patst*	A stock of citation-weighted patents applied by the firm in period *t*.
*Patst_gr*	A stock of citation-weighted patents granted by the patent authority among patents applied by the firm in period *t*.
(Control variable)	
*Pre_pat*	Dummy variable: 1 if the founder has experience of patent applications, 0 otherwise.
*Size*	Logarithm of the number of employees in the first year of observation.
*Age_firm*	Number of years after the foundation for the firm in period *t*.
*Age_founder*	Founders’ age at founding.
*Edu_univ*	Dummy variable: 1 if the founder had a university education before founding, 0 otherwise.
*Edu_X*	Dummy variable: 1 if the educational background of the founder is unknown, 0 otherwise.
(Instrumental variable)	
*Appro*	Industry’s degree of appropriability of innovation outcomes.
*Tech*	Industry’s degree of technological opportunities.

## Data

### Data sources

The data set comes from *COSMOS2*, compiled by Teikoku Databank Ltd. (TDB), a major credit reporting company in Japan. *COSMOS2* mainly collects information on incorporated firms, such as joint-stock companies, rather than sole proprietorships and partnerships. It provides basic information on founder- and firm-level characteristics, such as founders’ educational background and dates of birth, number of employees, and industry codes.^11^ It also provides information on whether a firm exits and its exit route.

The data set consists of 5270 joint-stock companies with fewer than 50 employees at the first year of observation in the manufacturing (3246 firms) and information services (software) sectors (2024 firms), founded between 2003 and 2010 and includes information on the survival and exit of firms from their foundation years to 2013.^12^ Some firms are regarded as censored before 2013 during the observation period, since TDB cannot track the firms’ information anymore (e.g., relocation for unknown reasons).^13^ In this study, we focus on joint-stock companies by excluding sole proprietorships and partnerships from the sample. Furthermore, this study targets new firms in the manufacturing and information services sectors because of their high R&D intensity and patent propensity.

To match the above data set with a patent database, we employ the *IIP Patent Database* compiled by the Institute of Intellectual Property (IIP), Japan Patent Office (JPO). This database covers all patent applications to the JPO since 1964.^14^ Using this database, we identify patent applications by firms based on their names and addresses.

Data on industry-specific characteristics used as instrumental variables, such as the appropriability of innovation output and technological opportunities, are obtained from the *Report on the National Innovation Survey 2003*, compiled by the National Institute of Science and Technology Policy (NISTEP) of the Ministry of Education, Culture, Sports, Science and Technology (MEXT).

### Descriptive statistics

Table 4 shows the life table for survival according to exit route by the year of foundation. The sample included eight cohorts from 2003 to 2010. Among them, there are approximately 500 firms founded between 2003 and 2005. The number of newly founded firms for each cohort increased from 2006 when a regulation for minimum capital requirement was abolished in Japan (Honjo & Kato, 2019).^15^ Many firms survived during the observation period and they were censored at the end of 2013. Among the 5270 firms in the sample, 624 (about 12%) had exited by 2013. The largest number of exits was voluntary liquidation (229) followed by bankruptcy (217) and merger (178). The exit rate in the sample seems much lower than found in previous studies (e.g., Dunne et al., 1988; Audretsch, 1995; Bartelsman et al., 2005). This is because we focus on joint-stock companies by excluding sole proprietorships and partnerships from the sample.

**Table 4:** Life table for survival according to exit route by the year of foundation

	Year of entry																															
	2003				2004				2005				2006				2007				2008				2009				2010			
		Exit events				Exit events				Exit events				Exit events				Exit events				Exit events				Exit events				Exit events		
Interval	*N*	*B*	*M*	*V*	*N*	*B*	*M*	*V*	*N*	*B*	*M*	*V*	*N*	*B*	*M*	*V*	*N*	*B*	*M*	*V*	*N*	*B*	*M*	*V*	*N*	*B*	*M*	*V*	*N*	*B*	*M*	*V*
2003–2004	482	0	2	0																												
2004–2005	480	3	3	1	490	0	0	1																								
2005–2006	471	4	2	4	489	2	2	0	538	0	0	0																				
2006–2007	458	3	6	8	485	5	4	4	537	5	0	2	866	0	0	1																
2007–2008	439	6	7	8	471	4	6	4	528	8	10	2	865	2	2	4	859	1	0	1												
2008–2009	412	9	5	7	453	8	5	4	503	7	5	8	854	5	5	8	857	4	5	3	937	0	0	0								
2009–2010	387	3	3	5	432	7	5	7	479	2	3	3	833	6	4	8	842	6	6	9	937	2	3	2	772	2	3	2				
2010–2011	373	4	3	2	413	3	2	1	468	10	3	2	808	7	9	9	816	6	3	12	925	4	5	7	765	2	0	8	326	0	1	7
2011–2012	360	2	0	3	404	6	3	2	451	4	4	2	777	9	5	2	790	7	5	5	901	3	3	4	747	8	5	9	315	0	3	9
2012–2013	355	5	4	0	392	2	1	1	441	2	5	1	753	7	4	5	767	4	4	7	885	4	3	7	715	9	4	5	297	5	3	13

Note: *N*, *B*, *M*, and *V* indicate the numbers of firms at risk, bankrupt firms, merged firms, and voluntarily liquidated firms, respectively. Some observations are censored during the observation period. However, the numbers of censored observations are not reported in this table for limited space

**Fig. 1:**
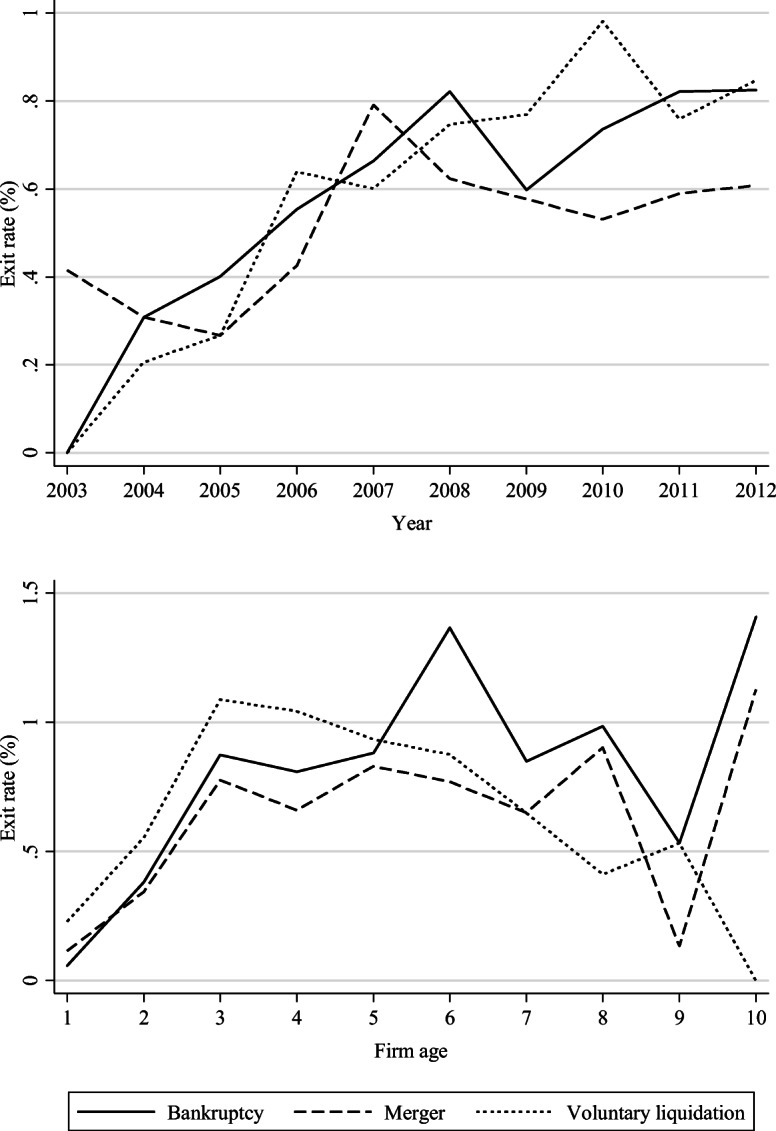
Exit rates according to observation years (upper) and firm age (lower)

Figure 1 describes the pattern of exits for each route during the observation period, according to year and firm age. The exit rate is defined as the number of exits over the number of firms at risk (shown as a percentage). In the upper figure, for example, the exit rate via merger is 0.4% in 2003 and zero for the other exit routes in the same year. As shown, the exit rates in all forms increase during the observation period.^16^ Similarly, the exit rates in all forms increase according to firm age, while those of voluntary liquidation peaked at the third year and then declined over time. While many studies have provided evidence that the probability of exit decreases with firm age, recent studies have indicated that age dependence is eliminated after controlling for firm quality, such as pre-entry performance and firm size (Thompson, 2005; Coad, 2018). Moreover, Fig. 1 seems consistent with the finding of Esteve-Pérez and Mañez-Castillejo (2008) who reported that the hazard rate increases as firms age up to 20 years, then declines up to 35 years, to finally increase again. In Japan, the bankruptcy rate is relatively low in the early years after foundation, partly because, as discussed in Section 2, bankruptcy takes a long time to complete.

Table 5 shows the summary statistics and correlation matrix of variables. The mean of a stock of citation-weighted patent application counts (*Patst*) is 0.358. The means of a stock of citation-weighted granted patent counts (*Patst_gr*) is 0.148, indicating that 41% of patent applications are granted for new firms in the sample. The value of *Patst* is more than zero in 2200 among 31,400 observations (7%), although that is not shown in this table. Similarly, the value of *Patst_gr* is more than zero in 1031 observations (3.3%). As for founders’ previous patent applications (*Pre_pat*), 318 founders (6%) in the sample had experience in patent applications before founding their firms. Furthermore, the average number of employees in the sample firms are approximately nine. The average age of founders at the time of founding is approximately 47 years.

**Table 5:** Summary statistics and correlation matrix of variables

Variable	Mean	Std.Dev.	(1)	(2)	(3)	(4)	(5)	(6)	(7)	(8)	(9)	(10)	(11)	(12)	(13)
(1) *Bankruptcy*	0.007	0.083	1												
(2) *Merger*	0.006	0.075	−0.006	1											
(3) *Voluntary*	0.007	0.085	−0.007	−0.007	1										
(4) *Patst*	0.358	2.906	0.005	−0.005	−0.003	1									
(5) *Patst_gr*	0.148	1.550	−0.005	−0.003	−0.005	0.867**	1								
(6) *Pre_pat*	0.061	0.239	−0.007	0.011*	0.014**	0.106**	0.080**	1							
(7) *Size*	1.792	1.037	0.018**	0.056**	−0.007	0.039**	0.023**	0.020**	1						
(8) *Age_firm*	3.837	2.237	0.035**	0.023**	0.009	0.073**	0.060**	0.007	0.087**	1					
(9) *Age_founder*	47.072	11.417	0.011*	0.014**	0.016**	0.043**	0.036**	0.155**	0.141**	−0.012**	1				
(10) *Edu*	0.414	0.493	−0.002	0.022**	0.008	0.041**	0.030**	0.056**	0.044**	0.056**	−0.044**	1			
(11) *Edu_x*	0.449	0.497	−0.010*	-0.009	0.007	−0.032**	−0.027**	−0.029**	−0.052**	−0.051**	0.065**	−0.760**	1		
(12) *Appro*	1.306	0.186	0.001	0.006	−0.001	0.052**	0.039**	0.124**	0.027**	0.001	0.134**	−0.009	−0.013**	1	
(13) *Tech*	0.949	0.127	−0.004	0.009	0.006	0.022**	0.012**	-0.003	−0.061**	0.045**	−0.284**	0.183**	−0.156**	−0.085**	1

Note: Number of observations is 31,400. ** and * indicate significance at the 1% and 5% levels, respectively

The correlation matrix of variables shows that *Patst* and *Patst_gr* are not significantly correlated to exit routes. *Pre_pat* is positively and significantly correlated to *Merger* and *Voluntary*. It is also positively correlated to *Patst* and *Patst_gr*, indicating that founders’ patenting experience affects the firms’ patent propensity. *Size* is positively and significantly correlated to *Bankruptcy* and *Merger*, while it is positively associated with *Patst* and *Patst_gr. Age_firm* is positively correlated to *Bankruptcy* and *Merger*, while it is positively associated with *Patst* and *Patst_gr.* These observations suggest that exit probabilities via bankruptcy and merger increase with firm size and age, and firms’ propensity to patent tends to increase with size and age. As for founder-specific characteristics, *Age_founder* is positively associated with all forms of exit and patent variables (*Patst* and *Patst_gr*). *Edu* is positively correlated to *Voluntary* and patenting variables. These observations suggest that we should take into account the endogeneity of patent variables in our model, since patenting is likely to be affected by a number of factors, such as founders’ patenting experience, firm size, and firm age. *Appro* and *Tech* used as instrumental variables are positively correlated to patent variables (*Patst* and *Patst_gr*), and the correlation coefficients are strongly significant at the 1% significance level. In contrast, these variables are not significantly correlated to all forms of exit. This indicates that the instrumental variables satisfy the conditions that they are not correlated to the exit decisions of firms as the dependent variables.

## Results

### Estimation results

Using the IV probit model, we estimated the effects of patenting on new firm survival according to exit route. The estimation results are shown in Table 6, when the stock of citation-weighted patent application counts (*Patst*) is used as the variable for patenting. The estimation results using the stock of citation-weighted granted patent counts (*Patst_gr*) are shown in Table 7. For both the tables, the results for the determinant of exit routes are shown in columns (i), (iii), and (v), while those from the first-stage regressions determining patenting are presented in columns (ii), (iv), and (vi). As seen in the bottom panels of Tables 6 and 7, the Wald tests of exogeneity of the instrumented variables indicate that the null hypothesis of no endogeneity, except for the case of voluntary liquidation as a route to exit in Table 6, is rejected. This means that the IV probit model should be used instead of a regular probit model.

**Table 6:** Estimation results for IV Probit regressions: effects of patent application stock (*Patst*) on exit routes

	Bankruptcy		Merger		Voluntary liquidation	
Variable	(i) *Bankruptcy*	(ii) *Patst*	(iii) *Merger*	(iv) *Patst*	(v) *Voluntary*	(vi) *Patst*
Endogenous variable						
*Patst*	−0.097***		0.212***		0.121	
	(0.012)		(0.000)		(0.077)	
Control variable						
*Pre_pat*	−0.063***	1.145***	−0.195***	1.145***	−0.009	1.145***
	(0.020)	(0.253)	(0.004)	(0.253)	(0.111)	(0.252)
*Size*	0.058	0.065	0.175***	0.065	−0.020	0.065
	(0.047)	(0.052)	(0.024)	(0.052)	(0.018)	(0.052)
*Age_firm*	0.063***	0.078***	0.010*	0.078***	0.025***	0.078***
	(0.013)	(0.021)	(0.005)	(0.021)	(0.001)	(0.021)
*Age_founder*	0.005	0.007	0.000	0.007*	0.004	0.007
	(0.003)	(0.004)	(0.000)	(0.004)	(0.004)	(0.005)
*Edu*	−0.154***	0.136**	0.260***	0.135**	0.314***	0.135**
	(0.005)	(0.065)	(0.013)	(0.065)	(0.015)	(0.064)
*Edu_X*	−0.189***	−0.011	0.213***	−0.011	0.317***	−0.011
	(0.002)	(0.150)	(0.035)	(0.150)	(0.031)	(0.150)
Instrumental variable						
*Appro*		0.588***		0.576***		0.581***
		(0.069)		(0.075)		(0.061)
*Tech*		0.590***		0.619***		0.606***
		(0.016)		(0.004)		(0.030)
Constant term	−2.767***	−1.740***	−2.500***	−1.756***	−2.867***	−1.749***
	(0.110)	(0.562)	(0.084)	(0.551)	(0.445)	(0.570)
Cohort dummies	Yes	Yes	Yes	Yes	Yes	Yes
Number of observations	31400		31400		31400	
Log pseudolikelihood	−78970.765		−78734.243		−79020.394	
Wald test of exogeneity (chi2)	37.46***		10180.00***		1.980	

Notes: Robust standard errors are in parentheses. ***, **, and * indicate significance at the 1%, 5%, and 10% levels, respectively

**Table 7:** Estimation results for IV Probit regressions: effects of granted patent stock (*Patst_gr*) on exit routes

	Bankruptcy		Merger		Voluntary liquidation	
Variable	(i) *Bankruptcy*	(ii) *Patst_gr*	(iii) *Merger*	(iv) *Patst_gr*	(v) *Voluntary*	(vi) *Patst_gr*
Endogenous variable						
*Patst_gr*	−0.248***		0.487***		0.263***	
	(0.084)		(0.036)		(0.058)	
Control variable						
*Pre_pat*	−0.074	0.458**	−0.170**	0.458**	0.003	0.458**
	(0.051)	(0.205)	(0.084)	(0.205)	(0.056)	(0.205)
*Size*	0.055	0.011	0.159***	0.012	-0.015	0.011
	(0.050)	(0.030)	(0.022)	(0.030)	(0.018)	(0.030)
*Age_firm*	0.063***	0.031***	0.008**	0.031***	0.025**	0.031***
	(0.013)	(0.009)	(0.004)	(0.009)	(0.012)	(0.009)
*Age_founder*	0.005*	0.003***	−0.001	0.004***	0.004*	0.004***
	(0.003)	(0.001)	(0.001)	(0.001)	(0.002)	(0.001)
*Edu*	−0.160***	0.029	0.237***	0.028	0.312***	0.029
	(0.017)	(0.031)	(0.085)	(0.032)	(0.052)	(0.031)
*Edu_X*	−0.196***	−0.032	0.197***	−0.032	0.313***	−0.032
	(0.004)	(0.084)	(0.069)	(0.084)	(0.013)	(0.084)
Instrumental variable						
*Appro*		0.228***		0.220**		0.224***
		(0.076)		(0.086)		(0.078)
*Tech*		0.187**		0.210**		0.199**
		(0.087)		(0.100)		(0.087)
Constant term	−2.754***	−0.604**	0.297**	0.431***	−2.186***	−0.618**
	(0.252)	(0.249)	(0.133)	(0.119)	(0.535)	(0.251)
Cohort dummies	Yes	Yes	Yes	Yes	Yes	Yes
Number of observations	31,400		31,400		31,400	
Log pseudolikelihood	−59358.017		−59125.116		−59409.003	
Wald test of exogeneity (chi2)	4.97**		9.31***		12.33***	

Notes: Robust standard errors are in parentheses. ***, **, and * indicate significance at the 1%, 5%, and 10% levels, respectively

As for the determinants of patenting in the first stage regression in Table 6, both *Appro* and *Tech* as (additional) instrumental variables have positive and significant effects on *Patst*.^17^*Pre_pat* has positive effects on *Patst*, indicating that firms whose founders have patenting experience are more likely to have a higher patent application stock.^18^*Age_firm* has positive and significant effects on *Patst*, indicating that the propensity to patent tends to rise with firm age. *Edu* has positive and significant effects on *Patst*, indicating that firms managed by founders with higher educational backgrounds are more likely to have a higher patent application stock. Turning to Table 7, the results of the first stage regressions are generally consistent with those of Table 6, while the coefficients and significance levels for founder-specific variables changed slightly.

Next, we discuss the effects of patenting on exit routes. With respect to *Bankruptcy*, column (i) of Table 6 shows the effect of the stock of citation-weighted patent applications (*Patst*) is negative and statistically significant at the 1% significance level, indicating that firms with a higher stock of patent applications are less likely to go bankrupt. Similarly, the effect of the stock of citation-weighted granted patents (*Patst_gr*) on *Bankruptcy* is negative and statistically significant at the 1% significance level in column (i) of Table 7, indicating that firms with a higher stock of granted patents are less likely to go bankrupt. Hypothesis 1 is supported with these findings. The findings suggest that patenting is an essential strategy to compete and survive successfully in product markets. It concurs with the RBV that patents are an important resource for new firms to protect inventions and attract customers and external providers of capital. As a result, patenting firms can obtain competitive advantage, thereby reducing the risk of business failure.

Turning to the effects of patenting on *Merger*, the effect of the stock of citation-weighted patent applications (*Patst*) is positive and statistically significant at the 1% significance level in column (iii) of Table 6, indicating that firms with a higher stock of patent applications are more likely to exit via merger. The effect of the stock of citation-weighted granted patents (*Patst_gr*) on *Merger* is positive and statistically significant in column (iii) of Table 7, indicating that new firms with a higher stock of granted patents are more likely to exit via merger. Our findings are consistent with those of previous studies, including Cotei and Farhat (2018), and Hypothesis 2 is supported. These findings suggest that patenting firms become more attractive targets for M&A by other firms. They also suggest that accumulating technological knowledge as well as holding patent rights as intangible assets are highly evaluated in markets for ideas. It concurs with the RBV that patents are a highly valuable resource in the markets.

As for the effects of patenting on voluntary liquidation, the stock of citation-weighted patent applications (*Patst*) has no significant effect on *Voluntary* in column (v) of Table 6. This indicates that new firms with a higher stock of patent applications do not necessarily opt for voluntary liquidation. As discussed in the previous section, only 41% of patent applications are granted in the sample as a result of examination in terms of novelty, usefulness, and non-obviousness. If firms get rejected for their patent applications, they are not able to hold patent rights and thus cash out their inventions in markets for ideas. As a result, such firms may not be different from non-patenting firms in terms of the propensity of voluntary liquidation. In contrast, the stock of citation-weighted granted patents (*Patst_gr*) has a positive and statistically significant effect on *Voluntary* in column (v) of Table 7, indicating that new firms with a higher stock of granted patents are more likely to voluntarily close their businesses without insolvency. In this respect, Hypothesis 3 is generally supported. The findings suggest the importance of patents as intangible assets in markets for ideas.

Regarding the effects of control variables, founders’ patent applications before founding (*Pre_pat*) have a negative and significant effect on the probability of exit via bankruptcy and merger in Table 6, while it is not significant in column (i) of Table 7, suggesting that new firms managed by founders with technological experience before founding are less likely to go bankrupt and exit via merger than those managed by founders without such experience. Firm size (*Size*) has a positive effect on exit via merger, as seen in Tables 6 and 7, indicating that larger firms are more likely to be targets of merger. Furthermore, firm age (*Age_firm*) has a positive effect on all forms of exit in Tables 6 and 7. The results indicate that the probability of exit, regardless of exit route, increases with age. While many studies have stated that the probability of exit decreases with firm age, our finding is consistent with the finding of Esteve-Pérez and Mañez-Castillejo (2008) that the hazard rate increases as the firm ages up to 20 years. As for founder-specific characteristics, founders’ age (*Age_founder*) has a positive and significant effect on exits via bankruptcy and voluntary liquidation in columns (i) and (v) of Table 7. Regarding the founders’ educational background, university education (*Edu*) has a negative and significant effect on the probability of bankruptcy, indicating that new firms managed by highly educated founders are less likely to go bankrupt. However, this variable has a positive and significant effect on the probability of merger and voluntary liquidation, indicating that new firms managed by highly educated founders are more likely to exit via merger or voluntary liquidation. These results are generally consistent with those of Kato and Honjo (2015).

### Robustness checks

We conduct some additional estimations to ensure the robustness of our findings. First, we estimate the model using a variable for the logarithm of current sales size in period *t*, since firm performance may be important to survival (e.g., Coad et al., 2013; Coad, 2018).^19^ The sample size is reduced due to some missing values (from 31,400 to 27,257). Table 9 in the Appendix shows the estimation results when the stock of citation-weighted granted patents (*Patst_gr*) is used as the variable for patenting. The results indicate that the effects of patenting are generally consistent with Table 7, while the current sales size significantly affects exit via voluntary liquidation.

Second, while the maximum age for a “new” firm in our sample is 10 years, previous studies have used different cut-off points in their definition of new firms; some have selected cut-off values of 6 or 8 years (Song et al., 2008; Cefis & Marsili, 2011). Therefore, we run the model with a restriction on the maximum firm age of 8 years^20^; the results are shown in Table 10 in the Appendix. These results are generally consistent with those shown in Table 7.

Third, we run a version of the model that excluded firms whose founders have experience in patent applications before founding to avoid an endogeneity problem, as such firms possess higher patent propensity; those results are shown in Table 11 in the Appendix and are generally consistent with those shown in Table 7.

## Discussion and conclusions

### Summary and contributions

This study examined the role of patenting in determining new firm survival according to exit routes. To do so, we constructed a large-scale sample of new firms in the Japanese manufacturing and information services sectors from 2003 to 2013. By distinguishing among bankruptcy, merger, and voluntary liquidation, this study shows sharp differences in the effects of patenting on new firm survival according to exit route. We found that while patenting lowers the probability of bankruptcy, it increases the probability of merger, regardless of patent measures used (applied or granted patent stock). In addition, we provided evidence that patenting increases the probability of voluntary liquidation only when granted patent stock is used as a variable for patenting. From the RBV, this study emphasizes the importance of patents as a valuable resource, ensuring competitive advantage in product markets and providing “bargaining chips” in markets for ideas.

This study contributes to the literature on the effects of patenting on firms’ exit. First, this study examined how the effects of patenting vary according to exit route, which is not well understood in the literature. As shown, there are sharp differences in the effects of patenting between the three routes to exit. Second, we estimated the model for the effects of patenting on exit routes while taking into account the endogeneity of patenting. Although previous studies, including Helmers and Rogers (2010), tend to ignore the endogeneity of patenting in their model, patenting is clearly a strategic decision for firms. By estimating the IV probit model, we clarified the effects of patenting on exit routes rather than simply showing correlations. Third, whereas previous studies have largely used applications as a measure of patenting, there is huge heterogeneity in quality among patents, and highly cited patents represent a particularly valuable resource (Wagner & Cockburn, 2010). By distinguishing between applied and granted patents (ex ante value of patents), as well as calculating patent stock adjusted by forward citations (ex post value of patents), we provided evidence on whether and how patent quality matters for new firm survival. In practice, while patent applications do not significantly affect voluntary liquidation, granted patents shows the significant effect on voluntary liquidation.

### Practical implications

This study has some practical implications. First, the findings suggest that accumulating high-quality patents can help new firms avoid bankruptcy in product markets, though acquiring and enforcing IP rights is costly, especially for small firms, due to large fixed costs (Lanjouw & Schankerman, 2004; Jensen & Webster, 2006). However, few new firms have their own IP professionals. Thus, from the perspective of economic policy, it is worth providing support for new firms’ IP acquisition and enforcement, while new firms should pay more attention to enhancing in-house knowledge and skills for IP management.

Second, as indicated in this study, patenting makes new firms more attractive as targets for merger, while it enables new firms to opt for voluntary liquidation as another successful exit strategy. While exit via M&A is generally the most successful case of cooperative strategies in markets for ideas, its likelihood is increased for new firms by patenting inventions. In addition, new firms can exit via voluntary liquidation as an alternative option in markets for ideas, even if new firms cannot find an appropriate partner for M&A. From an economic policy perspective, the markets for ideas, especially ones for M&As of new innovative firms, are not well developed in Japan; they should be further developed to provide entrepreneurs with more incentives to expand their businesses, especially high-tech ones. If new firms can conduct research and develop new knowledge more efficiently than large established firms, advancing such a “division of innovative labor” between new and large established firms will promote innovation and productivity growth (Arora et al., 2001).

Third, Japan has faced a low start-up rate (Honjo, 2015) and low economic growth over a long period of time (Fukao & Kwon, 2006). To achieve economic growth through entrepreneurship, Japanese policymakers have focused on creating more firms. For instance, the Abe administration’s growth strategy set a numerical target of a 10% start-up rate per year in Japan. However, some scholars have criticized this policy direction, arguing that it may promote the entry of low-productivity and non-innovative “revolving door” firms (e.g., Shane, 2009; Branstetter et al., 2014). As shown in this study, new firms acquiring high-quality patents tend to achieve better post-entry performance than other firms (e.g., lower probability of bankruptcy or higher probability of M&A). To promote sustainable economic growth, policymakers—especially in countries facing economic conditions similar to those in Japan—should shift their attention to the creation of innovative firms (Schneider & Veugelers, 2010; Colombelli et al., 2016).

### Limitations and future avenues of research

This study has some limitations. First, while this study sheds light on the exit routes of new firms, initial public offering (IPO) as entrepreneurial exit is not considered. IPO as well as M&A is an important option of exit for entrepreneurs (DeTienne & Cardon, 2012). Especially in Japan, IPO is much more common than M&A as a successful entrepreneurial exit (Honjo & Nagaoka, 2018; Honjo, 2020). It may be worthwhile extending the analysis to entrepreneurial exit, including IPO. Second, there may be concerns about the external validity of our findings. For example, this study focused only on newly founded joint-stock companies in Japan and did not include sole proprietorships in the sample. In addition, the institutional context in Japan may differ from that of other countries, which may affect the nature of the exit routes. For example, some scholars show that bankruptcy law in Japan is not entrepreneur-friendly compared to other countries, and it affects the exit intensions of new firms (Peng et al., 2010). Further analysis including sole proprietorships and using data from other countries is warranted.

Future avenues of research are suggested. First, while we focused on the patenting activity of new firms, it would be worthwhile to consider other measures of innovation activities, such as marketing and organizational innovations (e.g., Cefis & Marsili, 2019). IP other than patents would be important for new firms. For example, acquiring trademarks as a marketing asset may be an important strategy, especially in non-technological sectors such as services industries (e.g., Mendonça et al., 2004; De Vries et al., 2017). Second, while we focused on survival and exit as a measure of post-entry performance of firms, it would be worthwhile to use alternative measures, such as growth and profitability (Freel, 2000; Helmers & Rogers, 2011; Stam & Wennberg, 2009). Third, the role of patents as intangible assets may change depending on the environment, such as the global financial crisis and COVID-19. Cefis and Marsili (2019) showed that innovation plays a more important role in firm survival, especially in times of crisis. Landini et al. (2020) suggest that intangibles strengthen firms’ resilience, i.e., the ability to cope with unexpected shocks. Further analysis taking into account changing environments would enhance our understanding of the role of patenting in firms’ survival and exit.

## Appendix

**Table 8:** Industry distribution of the sample used in this study

			*N* of exits		
Industry	*N* of obs.	*N* of firms	Bankruptcy	Merger	Voluntary liquidation
Food, beverage, and feed	2952	528	25	14	23
Textiles	212	39	0	2	1
Apparel	555	101	4	4	4
Lumber and wood products	458	78	4	4	1
Furniture and product	354	64	3	3	2
Pulp, paper, and paper products	244	39	0	0	1
Publishing and printing	1419	249	17	15	7
Chemicals	701	122	4	11	6
Petroleum and coal products	73	12	1	1	1
Rubber products	194	30	1	0	0
Leather, leather products, and fur skins	163	30	3	0	0
Ceramic, stone, and clay products	807	131	4	7	5
Steel and non-ferrous metals	475	80	3	7	2
Fabricated metals	1916	338	9	7	14
General machinery	3293	563	27	13	23
Electrical machinery	1921	320	15	9	19
Transportation machinery	526	89	2	2	4
Precision machinery	500	84	8	2	3
Miscellaneous manufacturing	2038	349	13	8	14
Information services (software)	12,599	2,024	74	69	99
Total	31,400	5,270	217	178	229

**Table 9:** Estimation results for IV Probit regressions with current sales size

	Bankruptcy		Merger		Voluntary liquidation	
Variable	(i) *Bankruptcy*	(ii) *Patst_gr*	(iii) *Merger*	(iv) *Patst_gr*	(v) *Voluntary*	(vi) *Patst_gr*
Endogenous variable						
*Patst_gr*	-0.520***		0.465***		0.288***	
	(0.004)		(0.047)		(0.031)	
Control variable						
*Pre_pat*	0.181**	0.520**	−0.171*	0.520**	−0.098	0.520**
	(0.080)	(0.242)	(0.100)	(0.242)	(0.060)	(0.242)
*Sales_t*	0.001	0.022**	0.081	0.022**	−0.055***	0.022**
	(0.020)	(0.009)	(0.051)	(0.009)	(0.004)	(0.009)
*Size*	0.016	−0.004	0.129***	−0.005	0.045***	−0.004
	(0.070)	(0.030)	(0.011)	(0.030)	(0.003)	(0.030)
*Age_firm*	0.025***	0.024**	−0.015***	0.024**	0.009	0.024**
	(0.002)	(0.010)	(0.000)	(0.010)	(0.010)	(0.010)
*Age_founder*	0.006*	0.004***	0.000	0.004***	0.006**	0.004***
	(0.003)	(0.001)	(0.002)	(0.001)	(0.003)	(0.001)
*Edu*	−0.062***	0.036	0.162	0.035	0.289***	0.036
	(0.008)	(0.028)	(0.120)	(0.029)	(0.025)	(0.029)
*Edu_X*	−0.108**	−0.028	0.107***	−0.028	0.302***	−0.028
	(0.045)	(0.093)	(0.022)	(0.093)	(0.008)	(0.093)
Instrumental variable						
*Appro*		0.258***		0.249**		0.257***
		(0.083)		(0.098)		(0.088)
*Tech*		0.246***		0.266***		0.247***
		(0.073)		(0.100)		(0.085)
Constant term	−1.740***	−0.754**	−2.473**	−0.766**	−2.628***	−0.755**
	(0.450)	(0.318)	(0.993)	(0.308)	(0.001)	(0.310)
Cohort dummies	Yes	Yes	Yes	Yes	Yes	Yes
Number of observations	27,257		27,257		27,257	
Log pseudolikelihood	−53063.830		−52946.929		−53058.960	
Wald test of exogeneity (chi2)	27.07***		6.51**		21.01***	

Notes: Robust standard errors are in parentheses. ***, **, and * indicate significance at the 1%, 5%, and 10% levels, respectively

**Table 10:** Estimation results for IV Probit regressions: a sample restricting the maximum age to 8 years

	Bankruptcy		Merger		Voluntary liquidation	
Variable	(i) *Bankruptcy*	(ii) *Patst_gr*	(iii) *Merger*	(iv) *Patst_gr*	(v) *Voluntary*	(vi) *Patst_gr*
Endogenous variable						
*Patst_gr*	−0.157**		0.526***		0.263***	
	(0.074)		(0.013)		(0.010)	
Control variable						
*Pre_pat*	−0.115***	0.454**	−0.197**	0.455**	0.012	0.455**
	(0.019)	(0.196)	(0.080)	(0.196)	(0.012)	(0.196)
*Size*	0.057	0.010	0.141***	0.010	−0.015	0.010
	(0.050)	(0.028)	(0.016)	(0.028)	(0.017)	(0.028)
*Age_firm*	0.079***	0.034***	0.014**	0.034***	0.043***	0.034***
	(0.017)	(0.009)	(0.007)	(0.009)	(0.011)	(0.009)
*Age_founder*	0.005	0.004***	−0.001***	0.004***	0.004	0.004***
	(0.003)	(0.001)	(0.000)	(0.001)	(0.003)	(0.001)
*Edu*	−0.142***	0.030	0.208***	0.029	0.304***	0.029
	(0.003)	(0.029)	(0.067)	(0.030)	(0.034)	(0.029)
*Edu_X*	−0.194***	−0.026	0.170***	−0.026	0.312***	−0.026
	(0.015)	(0.078)	(0.059)	(0.078)	(0.001)	(0.078)
Instrumental variable						
*Appro*		0.208***		0.196**		0.203***
		(0.069)		(0.079)		(0.069)
*Tech*		0.191***		0.217***		0.203***
		(0.068)		(0.079)		(0.063)
Constant term	−2.884***	−0.583**	−1.948***	−0.597**	−2.815***	−0.590**
	(0.210)	(0.255)	(0.416)	(0.259)	(0.099)	(0.262)
Cohort dummies	Yes	Yes	Yes	Yes	Yes	Yes
Number of observations	30,293		30,293		30,293	
Log pseudolikelihood	−56882.582		−56670.977		−56956.336	
Wald test of exogeneity (chi^2^)	3.20*		15.10***		72278.45***	

Notes: Robust standard errors are in parentheses. ***, **, and * indicate significance at the 1%, 5%, and 10% levels, respectively

**Table 11:** Estimation results for IV Probit regressions: a sample dropping firms with *Pre_pat*=1 from the sample

	Bankruptcy		Merger		Voluntary liquidation	
Variable	*(i)Bankruptcy*	*(ii) Patst_gr*	*(iii) Merger*	*(iv) Patst_gr*	*(v) Voluntary*	*(vi) Patst_gr*
(Endogenous variable)						
*Patst_gr*	−0.114**		0.552***		0.496***	
	(0.045)		(0.036)		(0.038)	
(Control variable)						
*Size*	0.061	0.013	0.157***	0.013	−0.017*	0.013
	(0.046)	(0.029)	(0.013)	(0.029)	(0.009)	(0.029)
*Age_firm*	0.060***	0.028***	0.006	0.028***	0.015***	0.028***
	(0.011)	(0.005)	(0.004)	(0.005)	(0.002)	(0.005)
*Age_founder*	0.005	0.003**	−0.001***	0.003**	0.003	0.003**
	(0.004)	(0.001)	(0.000)	(0.001)	(0.004)	(0.001)
*Edu*	−0.170***	0.029	0.264***	0.028	0.302***	0.028
	(0.026)	(0.031)	(0.011)	(0.032)	(0.043)	(0.031)
*Edu_X*	−0.204***	−0.061	0.221***	−0.061	0.316***	−0.061
	(0.007)	(0.101)	(0.012)	(0.100)	(0.084)	(0.100)
(Instrumental variable)						
*Appro*		0.268***		0.259***		0.262***
		(0.029)		(0.032)		(0.025)
*Tech*		0.194***		0.222***		0.213***
		(0.019)		(0.029)		(0.009)
Constant term	−2.854***	−0.629**	−2.229***	−0.649**	−2.416***	−0.643**
	(0.177)	(0.257)	(0.023)	(0.254)	(0.458)	(0.266)
Cohort dummies	Yes	Yes	Yes	Yes	Yes	Yes
Number of observations	29,484		29,484		29,484	
Log pseudolikelihood	−49730.704		−49461.951		−49716.692	
Wald test of exogeneity (chi2)	10.62***		445.07***		26.98***	

Notes: Robust standard errors are in parentheses. ***, **, and * indicate significance at the 1%, 5%, and 10% levels, respectively.

## Footnotes

1 For a survey of the relationship between innovation and performance, see Rosenbusch et al. (2011).

2 Licensing-out using patents may be an effective strategy for new firms (Motohashi, 2008). Patenting firms can earn royalties by licensing out technologies to others; thus, they are less likely to go bankrupt. In this study, however, licensing-out is not analyzed explicitly, because it is not included in firm exit.

3 In this study, we focus on firm exit and do not include acquisitions in our empirical analysis. With respect to acquisition cases, an acquiring firm and an acquired firm are regarded as surviving firms because neither firm disappears, although ownership is transferred.

4 Narin et al. (1987) showed that patents are an excellent indicator of a firm’s overall technological strength.

5 Jensen and Webster (2006) presented evidence that smaller firms have a higher rate of IP usage, suggesting that patenting is an important strategy for small firms.

6 Serrano and Ziedonis (2019) showed evidence that most patents from failed start-ups are sold quickly, and remain alive through renewal fee payment long after the closure of these start-ups.

7 The European Patent Office (EPO) does not grant patents for computer programs (“software patents”) or computer-implemented business methods that make no such technical contribution (European Patent Office, 2013). In Japan, software was made eligible for patent protection in 2000, and this protection was extended to software that circulates on computer networks in 2002 (Kani & Motohashi, 2011). There are significant differences in the propensity to patent between the manufacturing and information services (software) sectors in our sample. The mean of a stock of citation-weighted patent application counts (this is defined later) is 0.390 in the manufacturing sector, while it is 0.310 in the information services sector. The mean is different at the 5% significance level (*t*-test).

8 Kubo and Saito (2012) reported that 31 of the 111 mergers involving Japanese companies listed between 1990 and 2003 were likely rescue mergers. According to Kang et al. (2000), rescue mergers represent instances where a bank, acting primarily as a creditor, forces a client firm to bail out another distressed firm for which the bank is also a creditor.

9 In this paper, technological area is defined using international patent classification (IPC) classes.

10 As of 2003, the rate of granted patents among total patent applications to the JPO was 49.9%, while it was 59.9% for the European Patent Office and 64% for the US Patent and Trademark Office (USPTO, 2004). Many patents applied for to the JPO have not been granted. According to Nagaoka (2009), one reason for the low grant rate may be that the JPO has applied stricter standards for patent quality since 2000, particularly regarding inventive steps. To grant patents in Japan, applicants need to request examination within 3 years from the filing date by the applicant or a third party, while there is no such a system in the USA. Another reason for the low grant rate in Japan might be because procedural costs are quite low at the time of patent application (van Pottelsberghe de la Potterie & François, 2009), but applicants are required to pay more than ten times those fees (168,000 yen) to request an examination, according to the JPO official webpage, in contrast to the application fees (15,000 yen) as of 2004.

11 As another data source, the *Establishment and Enterprise Census* reports data such as the number of entries and exits at the establishment level for individual industries or regions. However, it is difficult to obtain data for individual firms from this data source and, generally, we could not use the source to identify which establishments (or firms) have become active or extinct. Additionally, reliance on the source is accompanied by the possibility that relocations of establishments to other regions might be recorded as exits even if the establishments remain in the market.

12 Table 8 in the Appendix shows the industry distribution in the sample used in this study.

13 As a robustness check, we estimated the model by excluding from the sample firms that are censored during the observation period. However, the results do not change significantly.

14 For more details on this database, see Goto and Motohashi (2007).

15 Under this amendment of the *Commercial Act*, enacted in April 1990, when a joint-stock company was newly founded in Japan, paid-in capital of at least 10 million yen was required at founding. This regulation was removed by the introduction of the new *Companies Act* in May 2006, which superseded the *Commercial Act*.

16 To control for macroeconomic conditions, we estimated the model by inserting year dummies. These variables did not affect significantly all forms of exit. Therefore, we do not report the results using year dummies.

17 The *F*-test for weak-instrument cannot be done in the IV probit model. Instead, we estimated an IV regression and found that the *F*-value in the first stage regression exceeds 10, suggesting that our instruments are not weak. We estimated the model using *Appro* only as an additional instrumental variable. The results are generally consistent with those of Tables 6 and 7.

18 This is consistent with the finding of Balconi and Fontana (2011) that successful inventors are more likely to be serial patentees.

19 We further estimated the model by including sales growth measured as the log-differences of sales at founding year and at period *t* divided by the number of years after foundation. This variable was not significant for all forms of exit; therefore, we do not report it.

20 The results using a sample restricting the maximum age to six years are also consistent with those using the full sample.
